# Editorial: Neural circuits underlying general anesthetics mediated consciousness changes

**DOI:** 10.3389/fncir.2023.1251970

**Published:** 2023-09-04

**Authors:** Cheng Zhou

**Affiliations:** Laboratory of Anesthesia and Critical Care Medicine, National-Local Joint Engineering Research Centre of Translational Medicine of Anesthesiology, West China Hospital of Sichuan University, Chengdu, China

**Keywords:** general anesthetics, consciousness, neural circuits, developmental brain, electroencephalograms (EEG)

The application of modern general anesthetics (GA) in the clinic has been more than 170 years, which has fundamentally changed clinical surgeries and/or invasive medical procedures (Bao et al., 2021, 2023). Despite the revolutionary contribution of general anesthesia to the clinic, the precise mechanisms of how general anesthetics induce their pharmacological actions are not completely understood (Bao et al., 2023; Luo et al., 2023). There are subtypes of general anesthetics with structured diversity; therefore, although all general anesthetics work on the central nervous system to induce a comprehensive state that is mainly characterized by reversible loss of consciousness, they interact with many varied molecular targets, such as GABA_A_ receptors, NMDA receptors and many ion channels (Chen et al., 2021; Zhang et al., 2023a,b). In recent decades, the researches on general anesthetics have focused on searching for critical molecules that involved in general anesthesia (Platholi and Hemmings, 2022; Yi et al., 2023). Therefore, as mentioned above, our knowledge about the modulatory effects of general anesthetics on molecular targets is accumulating; but these molecules cannot be affected by all kinds of general anesthetics, and they even play different roles in differential brain regions during general anesthetic-mediated consciousness changes.

More recently, an increasing number of studies have focused on how the effects of general anesthetics on molecular targets can explain their pharmacological actions in neural circuits and/or at the systemic level (Hua et al., 2020; Cao et al., 2023; Luo et al., 2023). Studies on neural circuits may help us learn more about GA's overall influence on the brain, as well as the possible essential neuronal pathways or circuits in general anesthesia, also allowing us to piece together a complete picture of the mechanism of GA-mediated consciousness changes. For this trend, some critical neural circuits have been identified as the neural substrates for general anesthetic-mediated consciousness changes (An et al., 2021; Wang et al., 2021; Yang et al., 2022; Luo et al., 2023; Soplata et al., 2023). For example, it has been reported that the dynamic modulations of the thalamocortical loop, cortical fragmentation and/or sleep-arousal network play important roles (Yang et al., 2022; Luo et al., 2023; Soplata et al., 2023). However, little is known about which nuclei and types of neurons in these brain regions are responsible for general anesthetic-mediated consciousness changes. Therefore, it is still necessary to investigate the links between the effects of general anesthetics on molecular targets and/or nuclei to neural circuits, which is also the main bottleneck for our understanding of anesthetic mechanisms.

Originally, for this Research Topic, we aim to study the neural circuit formation and alterations associated with general anesthetic-mediated consciousness changes, as well as to identify nuclei and brain regions that are responsible for action of GA. Four papers have been included until now. The first research article (*Connection input mapping and 3D reconstruction of the brainstem and spinal cord projections to the CSF-contacting nucleus*) by Song et al. first illustrated the broad projections of the CSF-contacting nucleus from the brainstem and spinal cord, which implies the complicated functions of the nucleus, especially for the unique roles of coordination in neural and body fluid regulation. Interestingly, the inputs from subcerebral brainstem and/or spinal cord sensory inputs may affect the arousal state of consciousness and then change anesthetic requirements (Breton-Provencher et al., 2021; Sherman and Usrey, 2021; Caminero and Cascella, 2023). Therefore, this study may indicate a new projection from the subcerebral region that is involved in the potency of general anesthetics. Another review article (*Effect of anaesthetics on functional connectivity of developing brain*) by Chen et al. summarizes the effect and mechanism of anesthesia on the rapid growth and development infant and neonate brain with fMRI through functional connectivity. It is possible to provide a new mechanism of neuronal modulations induced by anesthetics and objective imaging evidence in the developing animal brain, which is an important concern for anesthetic application in pediatric anesthesia. Next, a research article (*Development of NMDA receptors contributes to the enhancement of electroencephalogram oscillations under volatile anaesthetics in rats*) by Zhang et al. followed the effects of general anesthetics on the developing brain. Electroencephalogram patterns under general anesthesia are the most important electrophysiological characteristics of the brain and are useful for both investigations of anesthetic mechanisms and anesthesia monitoring. This study indicates that volatile anesthetics, including sevoflurane and isoflurane, enhance oscillations in cortical electroencephalograms (EEGs), partly by modulating glutamate-mediated excitatory synaptic transmission. The development of NMDA receptors may contribute to the enhancement of cortical EEG oscillations under volatile anesthetics. Therefore, understanding the specific electroencephalogram patterns under general anesthesia in the developing brain is significant for monitoring the neuronal brain under anesthesia or can be used as a tool to understand brain development in turn. The last research article (*The volatile anesthetics isoflurane differentially inhibits voltage-gated sodium channel currents between pyramidal and parvalbumin neurons in prefrontal cortex*) by Qiu et al. investigated the effects of isoflurane on Na_v_ between parvalbumin (PV^+^) and pyramidal neurons in the mouse prefrontal cortex. As a result, isoflurane differentially inhibits Na_v_ currents between pyramidal and PV^+^ neurons in the prefrontal cortex, which may contribute to the preferential suppression of glutamate release over GABA release, resulting in the net depression of excitatory-inhibitory circuits in the prefrontal cortex. Therefore, this study provides a primary effect of the volatile anesthetic isoflurane on cortical circuits, which may help to explain how volatile anesthetics suppress cortical function and induce neuronal synchronization during anesthesia.

In summary, although there are currently only four articles included in the topic issue, all these articles describe a significant aspect of modulations of general anesthetic on neural circuits. Hopefully, there will be more researches on the actions of general anesthetics; and finally explain how general anesthetics induce consciousness changes.

## Author contributions

CZ wrote the paper.
